# Antibody response to Influenza booster vaccination in Franches‐Montagnes stallions supplemented with Equi‐Strath^®^: a randomized trial

**DOI:** 10.1002/vms3.95

**Published:** 2018-02-27

**Authors:** Hendrika A. van Dorland, Reto Zanoni, Vinzenz Gerber, Elise Jeannerat, Danja Wiederkehr, Dominik Burger

**Affiliations:** ^1^ School of Agricultural, Forest and Food Sciences Bern University of Applied Sciences Zollikofen Switzerland; ^2^ Institute of Virology and Immunology Vetsuisse Faculty University of Bern Bern Switzerland; ^3^ Swiss Institute of Equine Medicine University of Bern, and Agroscope Avenches Switzerland

**Keywords:** equine, plasmolyzed yeast, vaccination, influenza, antibody titre

## Abstract

Bio‐Strath^**®**^ is a plasmolyzed yeast product enriched with herbs, malt, honey and orange juice. In this study, the effect of Equi‐Strath^®^, the adapted product for horses, on the equine immune system was evaluated. A routine influenza booster vaccination was used as a model to study the effects of Equi‐Strath^®^ supplementation on the immune response. Twenty healthy Franches‐Montagnes stallions with pre‐existing antibody levels were randomly divided into a study group (SG,* n* = 10) receiving 0.06 mL/kg bodyweight of Equi‐Strath^**®**^, and a control group (CG,* n* = 10), receiving the same amount of placebo, daily. The supplement and placebo were given from week 1 until week 14 of the trial. After 10 weeks, the horses were vaccinated with a commercial vaccine containing equine influenza strains of the H3N8 subtype. Antibody titres in blood were measured at day 0 before vaccination, and 14 and 32 days after vaccination. In addition, a complete blood count (CBC) was performed on day 0 and day 32. A linear increase of haemagglutination inhibition titres in both groups was observed after vaccination, but with no difference between treatment groups. CBC components remained unaffected by treatment. In conclusion, daily Equi‐Strath^®^ supplementation did not affect the adaptive immune response in stallions after a routine commercial H3N8 influenza booster vaccination.

## Introduction

Successful equine influenza vaccination in horses stimulates an immune response, resulting in an increase of the antigen‐specific antibody production (Van Maanen & Cullinane 2002). The extent of the immune response, however, may be affected by several factors. Folsom *et al*. (2001) observed how exercise alters the immune response to equine influenza virus and may increase susceptibility to infection in ponies. In addition, Horohov *et al*. (1999) concluded that older horses had a reduced immune function, but were nevertheless more resistant to exercise‐induced immune suppression than younger horses. Dietary effects on the immune response in horses have also been reported. For instance, it was shown by several groups that dietary consumption of polyunsaturated fatty acids (PUFAs) can positively affect the immune system in horses (Curtis *et al*. 2000; Adam *et al*. 2003). Furthermore, a greater antigen‐specific antibody response to equine influenza was found in mature horses supplemented with a fourfold increased amount of vitamin E (Baalsrud & Overnes 1986). Knight & Tyznik (1990) observed a higher antibody response in vitamin E supplemented ponies challenged with packed sheep red blood cells intramuscularly compared to ponies supplemented with low levels of vitamin E. However, there are also reports with dietary supplements that show unaffected influenza vaccination responses (Brummer *et al*. 2013) or decreasing immunoglobulin concentrations in response to vaccination (Koke *et al*. 2015).

Equi‐Strath^®^ (Bio‐Strath AG, Zürich, Switzerland) for horses is, similar as Bio‐Strath^®^, a food supplement comprised of plasmolyzed herbal yeast, malt, honey and orange juice. Several studies have shown positive effects of this supplement on the immune system *in vivo* and *in vitro*. Leslie *et al*. (1988, 1989) showed that daily administration of Bio‐Strath^**®**^ prolonged survival time in old mice, even after infection with *Staphylococcus aureus*. As mortality rates in mice infected with *Staphylococcus aureus* were reduced and no changes in morbidity were observed, it was suggested that Bio‐Strath^**®**^ had a stress protectant effect (Leslie *et al*. 1988). In a study concerning the mode of action yielding higher survival rates, it could be determined that Bio‐Strath^**®**^ administration encourages more rapid and more efficient mobilization of leucocytes including helper T‐lymphocytes as well as B‐lymphocytes in mice (Joller 1988; Joller & Aeppli 1989). Similar findings were seen in T‐lymphocytes activation *in vitro* under the stress of low‐gravity as part of space biological investigations. The addition of the food supplement partly restored or even increased expression of activation markers on T‐lymphocytes after low‐gravity exposure (Schwarzenberg *et al*. 2000). At present, no scientific evidence is available illustrating the effects of Equi‐Strath^**®**^ on the horses’ health. The present study examined the effect of Equi‐Strath^**®**^ supplementation on antibody production and haematological parameters in response to a H3N8 influenza booster vaccination in healthy Franches‐Montagnes stallions.

## Material and methods

### Animals

The experiment was carried out at the Swiss National Stud in Avenches, and included 20 stallions of the Franches‐Montagnes breed. All horses used for the study were clinically healthy at the start of the experiment and had pre‐existing antibody titres. The stallions were on average 9.5 ± 4.5 years old, and had a bodyweight of 525 ± 30 kg (mean ± standard deviation). All of them were primary vaccinated and had received booster vaccinations every 6 months with the same strain as used in the current study. The stallions were kept in individual box stalls with straw or wood chip bedding and were fed hay or haylage. In addition, oats, barley, corn and pellets were fed daily. Water and a mineral salt block were available *ad libitum*. All animals were regularly exercised and were turned out daily into a paddock throughout the study.

### Experimental design

The stallions were randomly divided into two groups (drawn by lot), a study group (SG, *n* = 10; 8.6 ± 4.9 years, mean ± standard deviation) and a control group (CG, *n* = 10; 10.5 ± 4.1 years, mean ± standard deviation). The animals of the study group each received daily a 0.06 mL/kg bodyweight of Equi‐Strath^**®**^ **,** orally. The control group (CG) received a placebo equivalent in consistency, taste and colour as Equi‐Strath^®^, at the same quantity daily. The feeding staff were blinded to the group allocation of the stallions.

The experiment lasted for 14 weeks starting in October 2013. The supplement and the placebo were given from the beginning of the study (week 0) until week 14 of the trial. This treatment was also used for a parallel study on the effects of Equi‐Strath^®^ supplementation on semen quality and quantity (Burger *et al*., manuscript in preparation).

### Vaccination, blood sampling and immunological assay

After 10 weeks of daily supplementation with Equi‐Strath^®^, all horses were vaccinated against equine influenza strains A/equine/South Africa/4/03 and A/equine/Newmarket/2/93 of subtype H3N8 (Equilis^®^ Prequenza ad. us. vet., MSD Animal Health GmbH, Lucerne, Switzerland) as part of their half‐yearly routine booster vaccinations which were performed with the same vaccine for the previous 18 months The vaccine was administered by intramuscular injection in the left side of the neck. Immediately before week 10 of supplementation, which represents day 0 of the present study, and twice after vaccination (days 14 and 32 after vaccination), blood was sampled and analysed in order to determine the titres of specific antibodies. All blood samples were collected from the left jugular vein, immediately centrifuged (4000*g* for 10 min) and the serum frozen at −20°C until analysis. Antibody titres were determined via the haemagglutination inhibition (HI) test using the European representative strain A/equine/Suffolk/89 of subtype H3N8. The HI test was performed on microtitre plates according to standard procedures (Office International des Epizootiés (OIE), 1996). In addition, a complete blood count was carried out (IDEXX Procyte Dx Haematology Analyser, IDEXX Diavet AG, Bäch, Switzerland).

### Statistical analysis

Titre data were log2 transformed, and subsequently analysed using the mixed models procedure (SAS Inst. Inc., Cary, NC) with repeated measures. Two age classes were formed with horses < 9 years being class 1, and horses ≥ 10 years being class 2. The model included the group (horses with Equi‐Strath^**®**^ vs. horses with Placebo), the sampling time‐point (day 14 and day 32 after vaccination) and their interaction, and age class as fixed effects. In addition, the titre before the vaccination (day 0) was used as covariable in the model to adjust for initial differences between the two groups. The animal was specified as a random effect to accommodate repeated measures of single individuals. The interaction effect was dropped from the model as this was not shown to be significant during initial analysis.

Data on changes in titre compared to the titre measured before vaccination (Day 0) were analysed with the general linear model (GLM) procedure (SAS Inst. Inc., Cary, NC), including group and age class as fixed effects in the model. The interaction was dropped from the model as this was shown to be not significant during initial analysis. Effects were significant if *P* ≤ 0.05. Model assumptions were checked using a graphical analysis of residuals and normality of errors and random effects were assessed based on qq‐plots. The Mann–Whitney U test was used to compare differences between the two groups (SG vs. CG) for blood count components on day 0 before the vaccination, and for the change in blood count components between day 0 and 32 after the vaccination.

## Results and discussion

### Antibody titres

**Figure 1:**
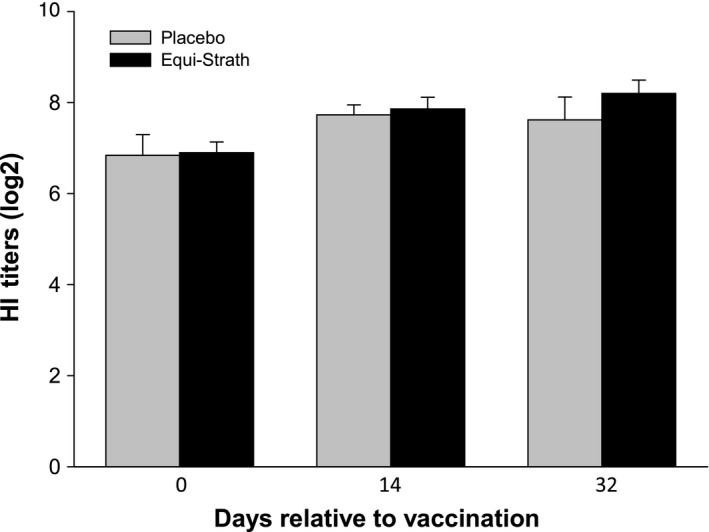
Haemagglutination inhibition titres (HI titres) of serum from stallions immunized with Equilis^®^ Prequenza ad. us. vet. vaccine over a 32‐day period during supplementation with Equi‐Strath^®^ or placebo. Results are means ± standard error of the mean (SEM).

According to studies in mice infected with *Staphylococcus aureus* (Joller 1988; Joller & Aeppli 1989), B‐lymphocytes and T‐lymphocytes are expected to be more efficiently and more rapidly activated after administration of Strath^**®**^ products. To evaluate the effect of Equi‐Strath^**®**^ on the immune system of healthy stallions, antibody titres were measured before and after H3N8 influenza vaccination. The results of the present study showed a numerically linear increase in haemagglutination inhibition titres in both groups after vaccination. However, no statistically significant effect (*P* = 0.66) of sampling time‐point was observed. In Gildea *et al*. (2011) as well as in other publications, it was shown that the immune response after vaccination peaks at 2 to 4 weeks. With regard to the treatment, slightly higher titres in response to vaccination were observed in horses supplemented with Equi‐Strath^®^ compared to the horses receiving the placebo, although the measured antibody titres of the stallions supplemented with Equi‐Strath^®^ did not differ significantly across time from those of the stallions supplemented with the placebo (*P* = 0.65; Figure 1). Likely, our influenza vaccination was not a sufficiently high and clinically relevant immune challenge to observe any effects from Equi‐Strath^®^ on the immune system, as was observed in the studies with mice infected with *Staphylococcus aureus* (Joller 1988; Joller & Aeppli 1989).

**Figure 2:**
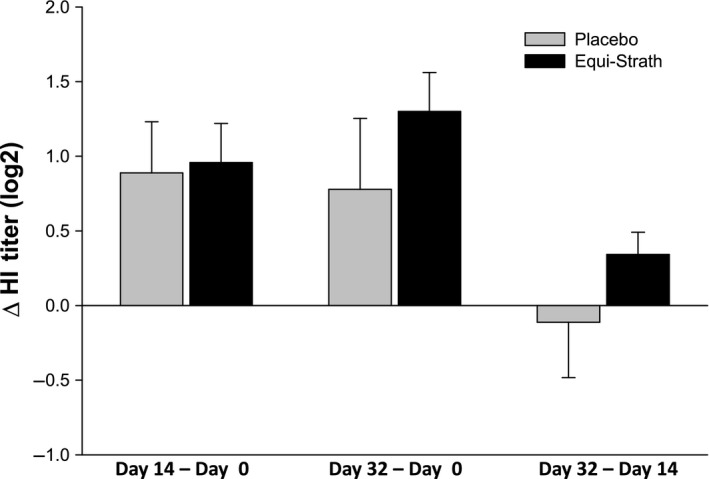
Change in the haemagglutination inhibition titres (HI titres, log2) of serum from stallions immunized with Equilis^®^ Prequenza ad. us. vet. vaccine over a 32‐day period during supplementation with Equi‐Strath^®^ or placebo.

The difference between day 14 to 0 and day 32 to 0 for means of titres (Δ HI; Figure 2) confirms a linear increase of the antibody titres over time, however, no significant effect of treatment was observed for any of the calculated differences. The same applies to the observed differences between day 14 to 32 being without a significant treatment effect.

Furthermore, we found an age class effect with younger horses having more markedly increased HI titres compared to older horses (*P* = 0.02) during the first 14 days after vaccination. Similarly Muirhead *et al*. (2010), showed age was a factor affecting the immune response, with aged horses having a significantly reduced response to influenza vaccination compared to the younger adult horses, likely because they had higher pre‐vaccination higher antibody titres. In their review, Solana & Pawelec (1998) question the use of booster vaccination in older horses as this may lead to a state of unresponsiveness due to ‘clonal exhaustion’ of the antigen‐specific lymphocytes. In the current study, Equi‐Strath^®^ supplementation did not modulate the immune response in stallions with respect to age.

### Complete blood count

The blood count did not show any significant difference between the study group and the control group over time from day 0 until day 32. Interestingly, at the day of vaccination, after 14 weeks of supplementation, significant differences between the groups in the WBC count, neutrophils, monocytes and eosinophils (Table 1) were found. The total WBC count was increased in the control group compared to the study group (*P* = 0.01). Also neutrophils (*P* = 0.02), monocytes (*P* = 0.03) and eosinophils (*P* = 0.01) were increased in the placebo control group compared to the study group. Even though, the observed differences were statistically significant, these small effects are not likely to be clinically relevant. As no blood samples for blood count were taken before the beginning of the administration of Equi‐Strath^®^ no comparison before and after the 14 week supplementation could be made.

**Table 1:** Blood count (mean ± SEM) in stallions supplemented with Equi‐Strath^®^ or placebo

Parameter	Days relative to vaccination	Experimental group		*P*‐value Mann–Whitney
		Equi‐Strath^®^	Placebo	
RBC (x10^12^/L)	0	7.29 ± 0.108	7.36 ± 0.115	0.48
Red blood cells	Changed 0–32	−0.10 ± 0.16	−0.06 ± 0.07	0.58
PCV (%)	0	32.66 ± 0.551	33.07 ± 0.518	0.41
Packed cell volume	Changed 0–32	−0.08 ± 0.76	0.14 ± 0.34	0.74
HGB (g/dL)	0	11.93 ± 0.156	12.06 ± 0.153	0.40
Haemoglobin	Changed 0–32	−0.15 ± 0.23	−0.04 ± 0.09	0.55
WBC (x 10^9^/L)	0	5.47 ± 0.277	6.56 ± 0.225	0.01
White blood cells	Changed 0–32	−0.20 ± 0.43	−0.33 ± 0.17	0.23
NEU (x10^9^/L)	0	3.05 ± 0.199	3.73 ± 0.172	0.02
Neutrophils	Changed 0–32	0.02 ± 0.27	−0.06 ± 0.12	0.63
LYM (x10^9^/L)	0	2.01 ± 0.090	2.25 ± 0.088	0.10
Lymphocytes	Changed 0–32	−0.19 ± 0.15	−0.26 ± 0.08	0.35
MONO (x10^9^/L)	0	0.27 ± 0.019	0.32 ± 0.015	0.03
Monocytes	Changed 0–32	−0.01 ± 0.03	0.00 ± 0.02	0.52
EOS (x10^9^/L)	0	0.12 ± 0.020	0.25 ± 0.047	0.01
Eosinophils	Changed 0–32	−0.01 ± 0.01	−0.02 ± 0.04	0.97
BASO (x10^9^/L)	0	0.019 ± 0.005	0.021 ± 0.004	0.70
Basophils	Changed 0–32	−0.00 ± 0.00	0.01 ± 0.01	0.11
PTL (K/*μ*L)	0	105.7 ± 11.09	124.1 ± 7.01	0.33
Platelet count	Changed 0–32	3.70 ± 9.89	−0.40 ± 11.20	0.85

## Conclusion

Daily Equi‐Strath^®^ supplementation did not affect the adaptive immune response in stallions after a routine H3N8 influenza vaccination. An effect of clinical relevance of Equi‐Strath^®^ on the immune system in horses was not demonstrated through the immunological stimulation by booster vaccination in horses with pre‐existing immunity. It remains to be elucidated, whether Equi‐Strath^®^ supplementation may induce a protective effect in horses exposed to a stronger immune challenge or in immunologically naïve horses without any previous vaccination.

## Source of funding

This work was supported by *ISMEquine Research, the School of Agricultural, Forest and Food Sciences (HAFL),* and by Bio‐Strath^**®**^.

## Conflict of interest

None of the authors has a financial or personal relationship with people or organizations that could inappropriately influence or bias this study.

## Ethical statement

The experiment followed the Swiss Law on Animal Protection and was authorized by the Committee of Animal Experiments of the Canton Vaud, Switzerland (no. 2667.1).

## Contributions

H.v.D, R.Z., V.G. and D.B. designed the study, D.B. supervised the experiments that were performed by R.Z., E.J. and D.W., H.v.D. analyzed the data. H.v.D., D.W. and D.B. wrote the manuscript that was then critically revised by all authors.

## Data access and responsibility

The principal investigators, Hendrika van Dorland and Dominik Burger, had full access to all of the data in the study and take the responsibility for the integrity of the data and the accuracy of the data analysis.

## Supporting information


**Table S1.** CONSORT 2010 checklist of information to include when reporting a randomized trial*.

**Figure S1.** CONSORT 2010 Flow Diagram.
