# Supplementary material for: Antibody response to Influenza booster vaccination in Franches‐Montagnes stallions supplemented with Equi‐Strath®: a randomized trial
Source: Vet Med Sci. 2018 Feb 27;4(2):133–9. doi: 10.1002/vms3.95 (PMC5980167; doi:10.1002/vms3.95)
Supplement: Supplementary file 2 — Figure S1. CONSORT 2010 Flow Diagram. [file VMS3-4-133-s002.doc]

**
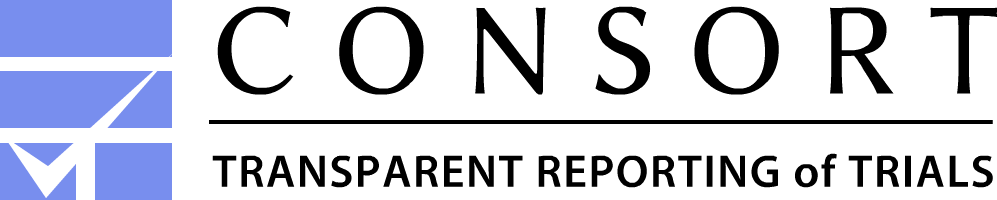
**

**CONSORT 2010 Flow Diagram**

**Allocation**

**Analysis**

**Follow-Up**

**Enrollment**

Assessed for eligibility (n=20)

Excluded (n=0)

♦  Not meeting inclusion criteria (n=0)

♦  Declined to participate (n=0)

♦  Other reasons (n=0)

Analysed (n=10)
♦ Excluded from analysis (give reasons) (n=0)

Lost to follow-up (give reasons) (n=0)

Discontinued intervention (give reasons) (n=0)

Allocated to intervention (n=10)

♦ Received allocated intervention (n=10)

♦ Did not receive allocated intervention (give reasons) (n=0)

Lost to follow-up (give reasons) (n=0)

Discontinued intervention (give reasons) (n=0)

Allocated to intervention (n=10)

♦ Received allocated intervention (n=10)

♦ Did not receive allocated intervention (give reasons) (n=0)

Analysed (n=10)
♦ Excluded from analysis (give reasons) (n=0)

Randomized (n=20)
